# Detecting functional field units from satellite images in smallholder farming systems using a deep learning based computer vision approach: A case study from Bangladesh

**DOI:** 10.1016/j.rsase.2020.100413

**Published:** 2020-11

**Authors:** Ruoyu Yang, Zia U. Ahmed, Urs C. Schulthess, Mustafa Kamal, Rahul Rai

**Affiliations:** a Department of Mechanical and Aerospace Engineering, 240 Bell Hall, University at Buffalo, Buffalo, NY, 14260-4400, USA; b Research and Education in Energy, Environment and Water (RENEW) Institute, 112 Cook Hall, University at Buffalo, Buffalo, NY, 14260-1300, USA; c CIMMYT-Henan Collaborative Innovation Center, Henan Agricultural University, Zhengzhou, 450002, PR China; d CIMMYT-Bangladesh, House 10/B, Road 53, Gulshan 2, Dhaka, 1212, Bangladesh

**Keywords:** Field boundaries, Smallholder farming, Deep learning, CNN

## Abstract

Improving agricultural productivity of smallholder farms (which are typically less than 2 ha) is key to food security for millions of people in developing nations. Knowledge of the size and location of crop fields forms the basis for crop statistics, yield forecasting, resource allocation, economic planning, and for monitoring the effectiveness of development interventions and investments. We evaluated three different full convolutional neural network (F–CNN) models (U-Net, SegNet, and DenseNet) with deep neural architecture to detect functional field boundaries from the very high resolution (VHR) WorldView-3 satellite imagery from Southern Bangladesh. The precision of the three F–CNN was up to 0.8, and among the three F–CNN models, the highest precision, recalls, and F-1 score was obtained using a DenseNet model. This architecture provided the highest area under the receiver operating characteristic (ROC) curve (AUC) when tested with independent images. We also found that 4-channel images (blue, green, red, and near-infrared) provided small gains in performance when compared to 3-channel images (blue, green, and red). Our results indicate the potential of using CNN based computer vision techniques to detect field boundaries of small, irregularly shaped agricultural fields.

## Highlights

• F–CNN based computer vision techniques were evaluated to detect boundaries in small, irregularly shaped fields.
• The DenseNet model performed best among three different F–CNN models, with F-1 scores for boundaries >0.8.
• The combination R-G-B bands with Near Infrared gave slightly better classification results than the R-G-B bands.

## Graphical abstract

**Figure:**
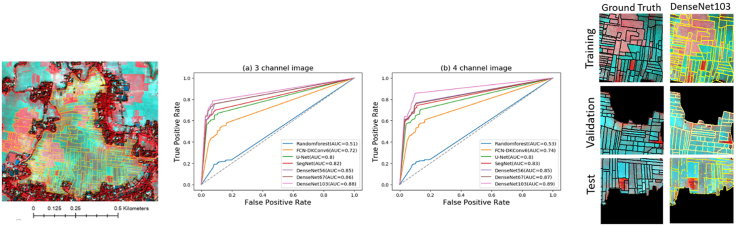

## 1 Introduction

Smallholder farms provide up to 90% of the food in developing nations (Singh, 2002). The sizes of smallholder farms are small, typically less than 2 ha. The land is split into several parcels, on which farmers sometimes grow a mix of different crops, and the boundaries are hard to distinguish (Fritz and See, 2008). As the human population keeps growing in many developing countries (Haub, 2013), field sizes will likely decrease, and more marginal lands will be brought into production (Debats et al., 2016). Given the potential role of smallholder agriculture in addressing food security, it is vital to gather spatial information of functional agricultural field units and how field units vary within and across geographic regions and over time. Such information is essential for improving crop yield prediction, providing crop management advice, resource allocation, economic planning, and monitoring the effectiveness of development interventions and investments. Many developing countries do not have an electronically accessible cadaster system in place. Moreover, field boundaries are not static.

Satellite data with a resolution up to 0.3 m open opportunities do delineate field boundaries, or functional agricultural field units, at a reasonable cost. Yet the development of an automatic field boundary detection and extraction method for smallholder farms across a broad range of agricultural environments is a complex challenge. Some studies have shown promising results (Debats et al., 2016; Yan and Roy, 2014). It is prudent to develop a specialized methodology using machine learning algorithms and readily available satellite images to tackle this non-trivial problem.

Early automatic and semi-automatic techniques for boundary delineation were based on edge detection methods such as Roberts detector (Roberts, 1963), Sobel edge detector (Gupta and Mazumdar, 2013), Laplacian of Gaussian detector (Gonzalez et al., 2004), and Canny edge detector (Canny, 1986). Edge detection combined with image segmentation (Pavlidis and Liow, 1990; Schoenmakers, 1995), graph-based vectorization (Turker and Kok, 2013), and multi-scale contrast limited adaptive histogram equalization (Graesser and Ramankutty, 2017) have been used for boundary detection. But the application of such methods tends to result in over-segmentation (Schick, 2011). They need parameter tuning through trial and error since they are highly dependent on a correct parameter selection (García-Pedrero et al., 2017). Recent agricultural field boundary detection techniques such as the line segment detection algorithm (LSD), the variational region-based geometric active contour method (VRGAC), when combined with a watershed segmentation algorithm, have performed reasonably well in regularly shaped agricultural fields (Alemu, 2016; Yan and Roy, 2014), yet failed to detect boundaries in heterogeneous landscapes dominated by smallholder farms. Moreover, due to their inherent complexity, these methods are not scalable over the broad range of agricultural landscapes where land cover displays high Spatio-temporal variability.

Recently, machine learning algorithms with deep neural architecture such as the convolutional neural network (CNN) have shown superior performances in object detection in a variety of imagery (Girshick et al., 2014; Krizhevsky et al., 2012; Ren et al., 2015). The availability of vast amounts of satellite imagery with the high spatial and temporal resolution has enabled several significant recent efforts to automate object detection, such as buildings (Gavankar and Ghosh, 2018; Yang et al., 2018a), roads (Xu et al., 2018a; Zhang et al., 2018), vehicles (Fan et al., 2016), airports (Xu et al., 2018b) and ships (Yang et al., 2018b; Zhang et al., 2016). Most of these methods were developed for object detection in urban settings, where regularly shaped objects dominate. They have not been adequately adapted or optimized for the detection of poorly delineated objects, such as small crop fields from satellite images (Ren et al., 2018). Only a few studies have adapted CNN for classification and extraction of crop fields from satellite imagery (Ji et al., 2018; Kussul et al., 2017; Zhong et al., 2017), and these studies have concentrated on large-scale farming applications. Recently, Musyoka (2018) applied F–CNN to detect agricultural field boundaries in northern Nigeria, where the average field size is 0.53 ha (FAO, 2019) and found that F–CNN outperformed other, traditional edge-detectors algorithms. Fully convolutional neural networks were able to accurately delineate boundary classes by learning the spatial-contextual features in a very complex dataset. In Bangladesh, the average size of landholding per farm is only about 0.3 ha. The land is split up into several parcels, resulting in areas of 0.08 ha for small and 0.16 ha for medium farms (Rahman and Rahman, 2009). This country represents one of the most challenging environments for automated field boundary detection from satellite images.

Semantic segmentation, a pixel-wise image recognition technique with deeper CNN architecture such as SegNet (Badrinarayanan et al., 2017), U-Net (Ronneberger et al., 2015), DenseNet (Huang et al., 2017), and RefineNet (Lin et al., 2017) might be a suitable technique for automated field boundary detection in this environment. It has been used in remote sensing applications for the detection of roads, buildings, water, and trees (Buslaev et al., 2018; Tao et al., 2018). In this study, we evaluated these three F–CNN based algorithms, U-net, SegNet, and DenseNet, to detect field boundaries from very high resolution (VHR) satellite images in a rice-based cropping system of Bangladesh. Also, two state-of-the-art pixel-wise segmentation methods named random forest (Debats et al., 2016) and FCN-DKConv6 (Musyoka, 2018) are also applied as the comparison method to verify the feasibility of proposed F–CNN based algorithms.

## 2 Material and methods

### 2.1 Study site

**Fig. 1:**
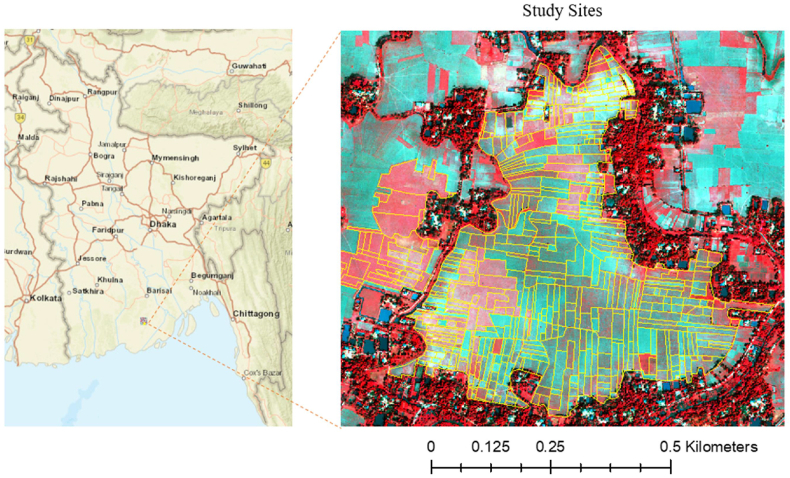
False-color R-G-B image of the study area with manually drawn field boundaries, Patuakhali District, Bangladesh.

We used a WorldView-3 multispectral image from the Patuakhali District, a southern coastal district in Bangladesh (Fig. 1), acquired on March 18, 2015. Its ground sampling distance (GSD) is 1.24 m. Agriculture in this region is characterized by a complex rice-based cropping system with small and fragmented fields. The study sites consist of 557 fields with an average field size of 0.105 ha. The fields are separated by paddy bunds (dikes), usually only about 0.3 m wide. In the winter season, which is dry, a large part of the study region is covered by low-intensity *rabi* crops (dry season crops) such as grass pea (*Lathyrus sativus* L.) and mung bean (*Vigna radiata* (L.) Wilczek) or left fallow. Some fields are cultivated with irrigated rice (*boro*).

### 2.2 Remote sensing and ground truth data

A very high resolution (VHR) WorldView-3 satellite image was obtained through the STARS project of the International Maize and Wheat Improvement Center (CIMMYT). The WorldView-3 sensor specifications are shown in Table 1. For this study, we used the blue, green, red, and near-infrared-2 bands.

**Table 1:** WorldView-3 sensor specifications.

Band	Wavelength (nm)	Sensor Resolution	Swath Width	Revisit Frequency (at 40°N Latitude)
Panchromatic	450–800	Panchromatic: 0.31 m GSD at nadir, 0.34 m GSD at 20° off-nadir Multispectral:	13.1 km at nadir	Less 1 day at 1 m GSD or 4.5 days at 20° off-nadir or less
				
8 Multispectral bands		1.24 m GSD at nadir, 1.38 m GSD at 20° off-nadir		
Coastal	400–450			
Blue	450–510			
Green	510–580			
Yellow	585–625			
Red	630–690			
Red Edge	705–745			
Near-IR1	770–895			
Near-IR2	860–1040			

**Fig. 2:**
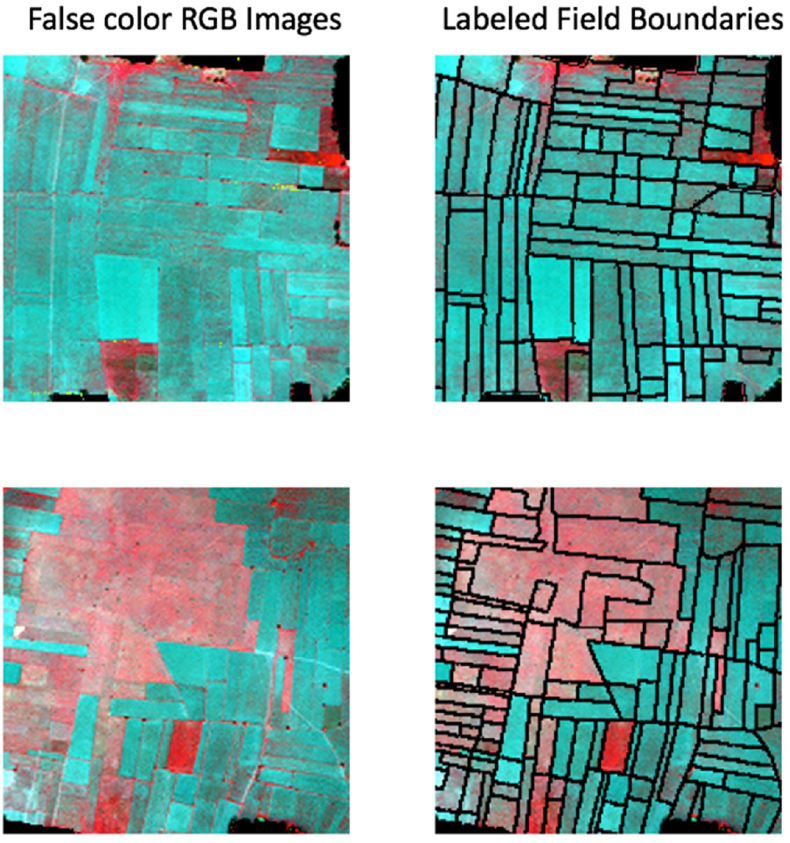
Sample false-color R-G-B images and corresponding labeled field boundaries.

We performed on-screen digitization in ArcGIS 10 (ESRI, 2019) to draw the boundaries of each field. We applied a buffer (0.5 m) function on the resulting single line vector data to create polygons of field boundaries. The resulting polygons were labeled as the boundary class (Class 1), and the region not covered by the buffer (the agricultural fields) labeled as the non-boundary class (Class 2). Finally, we converted this vector polygon to a binary raster format at 0.5 m resolution (Fig. 2).

### 2.3 Preprocessing

**Fig. 3:**
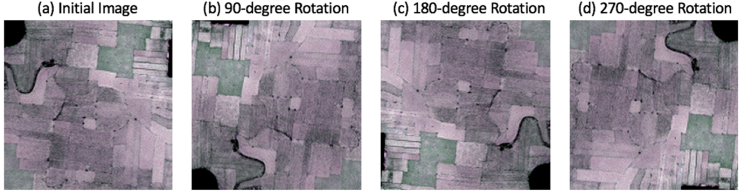
Data augmentation (a) initial Image (b) 90-degree rotation (c) 180-degree rotation (d) 270-degree rotation.

Deep learning algorithms are “data-hungry” as they work better with a large amount of data, and deep learning models trained with small datasets do not generalize well. Data augmentation techniques can be used to create additional data by modifying the original data without changing their meaning (Perez and Wang, 2017; Simard et al., 2003) and reducing the variance of the model and overfitting (Krizhevsky et al., 2012). There are several data augmentation methods available in computer vision-based image processing techniques such as horizontal/vertical flip, rotation, color modification, noise addition, size modification, and affine transformation. Before augmentation, we split both the very high resolution (VHR) and the labeled raster images containing the boundary (Class 1) and field classes (Class 2) into 51 images of 192 × 192 pixels. Then we applied 90-, 180- and 270-degree rotations on all images (Fig. 3) and generated a total of 204 image tiles. These images were split into 172 training, 8 validation, and 24 test images.

### 2.4 Boundary detection methods

We evaluated three different full convolutional neural networks (F–CNN) models, such as U-Net, SegNet, and DenseNet)with deep neural architecture to detect functional field boundaries from the very high resolution (VHR) WorldView-3 satellite imagery. We also used random forest (RF) and FCN-DKConv6 as base models to evaluate the performance of the proposed three kinds of F–CNN models.

#### 2.4.1 F–CNN based methods

Data-driven machine learning methods, especially CNN, have recently been widely used in the domain of remote sensing (Fan et al., 2016; Gavankar and Ghosh, 2018; Yang et al., 2018b). Although deep CNNs are very successful in object classification, their performance degrades when faced with semantic segmentation tasks such as field boundary detection. Due to a loss of object details at the pixel level, most of the deep CNN cannot recognize the specific object contour and fail to provide the right classification label to each pixel. This weakness can be overcome by a classification model that uses fully connected layers to predict the classes. The F–CNN model substitutes the last fully connected layer with a convolutional layer to capture the global context of the image. For field boundary detection, F–CNN is advantageous over CNN architectures for two reasons: First, an F–CNN naturally operates on an input of any size and produces an output of corresponding (possibly resampled) spatial dimensions (Long et al., 2015). Second, F–CNN is more efficient. They avoid tedious convolution computations and memory storage problems. In general, the encoder-decoder architecture is the most popular F–CNN model. The encoder can gradually reduce the input dimension, and the decoder gradually restores the details of the objects and the spatial dimension. Commonly, skip connections between the encoder and the decoder are used to enable better restoration details. In this study, we tested the suitability of three existing F–CNN based semantic segmentation architectures, namely U-Net, SegNet, and DenseNet, to detect field boundaries from VHR satellite images.

##### 2.4.1.1 U-Net

**Fig. 4:**
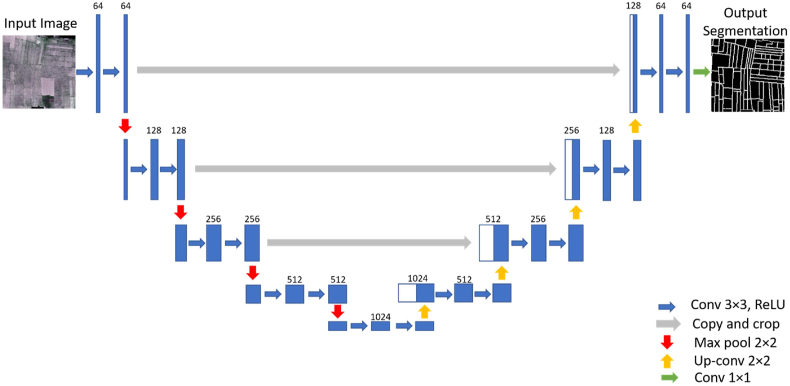
The architecture of the proposed U-Net model for satellite image segmentation.

U-net is a class of a fully convolutional network with symmetrical encoder-decoder deep learning architecture developed explicitly for biomedical image segmentation (Ronneberger et al., 2015). The encoder part is the typical convolutional neural network where each step consists of two 3 × 3 convolutional layers followed by a rectified linear unit (ReLU) and a 2 × 2 max pooling layer with stride 2. Each step of the decoder part involves the two 3 × 3 convolutional layers followed by a 2 × 2 up-convolutional layer. The cropping is necessary due to the loss of border pixels in every convolution. The last layer is a 1 × 1 convolutional layer for mapping from the feature vectors to the number of classes (Ronneberger et al., 2015). U-Net does not have any fully connected layers and only uses the valid part of each convolution that allows for the seamless segmentation of randomly large images. It uses an overlap-tile strategy replacing pooling operators with up-sampling operators. U-Nets can learn efficiently with low to medium quantities of training data and have recently been used for satellite image segmentation and object detection (Buslaev et al., 2018; Chhor and Aramburu, 2017; Rakhlin et al., 2018). The architecture and parameters of the U-Net architecture used for field boundary detection are shown in Fig. 4 and Table 2.

**Table 2:** Parameters of U-Net architecture (k: kernel size, c: channel number).

Layer	Down-sampling	Layer	Up-sampling
Conv1	k (3 × 3)/c (64)	Upsampling1	k (2 × 2)
Conv2	k (3 × 3)/c (64)	Conv11	k (3 × 3)/c (512)
Dropout1	0.5	Conv12	k (3 × 3)/c (512)
Maxpool1	k (2 × 2)	Dropout5	0.5
Conv3	k (3 × 3)/c (128))	Upsampling2	k (2 × 2)
Conv4	k (3 × 3)/c (128))	Conv13	k (3 × 3)/c (256)
Dropout2	0.5	Conv14	k (3 × 3)/c (256)
Maxpool2	k (2 × 2)	Dropout6	0.5
Conv5	k (3 × 3)/c (256)	Upsampling3	k (2 × 2)
Conv6	k (3 × 3)/c (256)	Conv15	k (3 × 3)/c (128)
Dropout3	0.5	Conv16	k (3 × 3)/c (128)
Maxpool3	k (2 × 2)	Dropout7	0.5
Conv7	k (3 × 3)/c (512)	Upsampling4	k (2 × 2)
Conv8	k (3 × 3)/c (512)	Conv17	k (3 × 3)/c (64)
Dropout4	0.5	Conv18	k (3 × 3)/c (64)
Maxpool4	k (2 × 2)	Dropout8	0.5
Conv9	k (3 × 3)/c (1024)	Conv19 (softmax)	k (1 × 1)/c (2)
Conv10	k (3 × 3)/c (1024)		

##### 2.4.1.2 SegNet

**Fig. 5:**
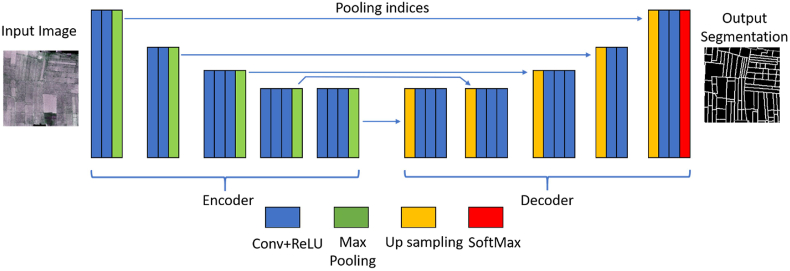
The architecture of the proposed SegNet model for satellite image segmentation.

SegNet (Badrinarayanan et al., 2017) is another symmetrical encoder-decoder deep learning architecture. Unlike U-net, it uses all pre-trained convolutional layer weights like Visual Geometry Group (VGG) net (Simonyan and Zisserman, 2014) as pre-trained weights in the decoding steps. However, a SegNet encoder network is smaller (only 13 layers) than VGG-net. Similar to the U-Net architecture, each encoder involves several convolutional layers followed by a rectified linear unit (ReLU) and a 2 × 2 max pooling layer with stride 2. For the decoder part, it upsamples its input feature map using the memorized max-pooling indices from the corresponding encoder feature map. This step produces a sparse feature map (Badrinarayanan et al., 2017). In the last layer of the decoder part, the softmax classifier is utilized to predict the label for each pixel in the input image. Moreover, by reusing max-pooling indices in the decoding process, SegNet reduces the number of parameters in the training process and performs well in boundary delineation and object detection from satellite images (Panboonyuen et al., 2017). The architecture and parameters of SegNet used for field boundary detection in our study are listed in Fig. 5 and Table 3.

**Table 3:** Parameters of SegNet architecture (k: kernel size c: channel number).

Layer	Down-sampling	Layer	Up-sampling
Conv1	k (3 × 3)/c (64)	Upsampling1	k (2 × 2)
Conv2	k (3 × 3)/c (64)	Conv14	k (3 × 3)/c (512)
Dropout1	0.5	Conv15	k (3 × 3)/c (512)
Maxpool1	k (2 × 2)	Conv16	k (3 × 3)/c (512)
Conv3	k (3 × 3)/c (128))	Dropout6	0.5
Conv4	k (3 × 3)/c (128))	Upsampling2	k (2 × 2)
Dropout2	0.5	Conv17	k (3 × 3)/c (512)
Maxpool2	k (2 × 2)	Conv18	k (3 × 3)/c (512)
Conv5	k (3 × 3)/c (256)	Conv19	k (3 × 3)/c (512)
Conv6	k (3 × 3)/c (256)	Dropout7	0.5
Conv7	k (3 × 3)/c (256)	Upsampling3	k (2 × 2)
Dropout3	0.5	Conv20	k (3 × 3)/c (256)
Maxpool3	k (2 × 2)	Conv21	k (3 × 3)/c (256)
Conv8	k (3 × 3)/c (512)	Conv22	k (3 × 3)/c (256)
Conv9	k (3 × 3)/c (512)	Dropout8	0.5
Conv10	k (3 × 3)/c (512)	Upsampling4	k (2 × 2)
Dropout4	0.5	Conv23	k (3 × 3)/c (128)
Maxpool4	k (2 × 2)	Conv24	k (3 × 3)/c (128)
Conv11	k (3 × 3)/c (512)	Dropout9	0.5
Conv12	k (3 × 3)/c (512)	Upsampling5	k (2 × 2)
Conv13	k (3 × 3)/c (512)	Conv25	k (3 × 3)/c (64)
Dropout5	0.5	Conv26	k (3 × 3)/c (64)
Maxpool5	k (2 × 2)	Dropout10	0.5
		Conv27 (softmax)	k (1 × 1)/c (2)

##### 2.4.1.3 DenseNet

**Fig. 6:**
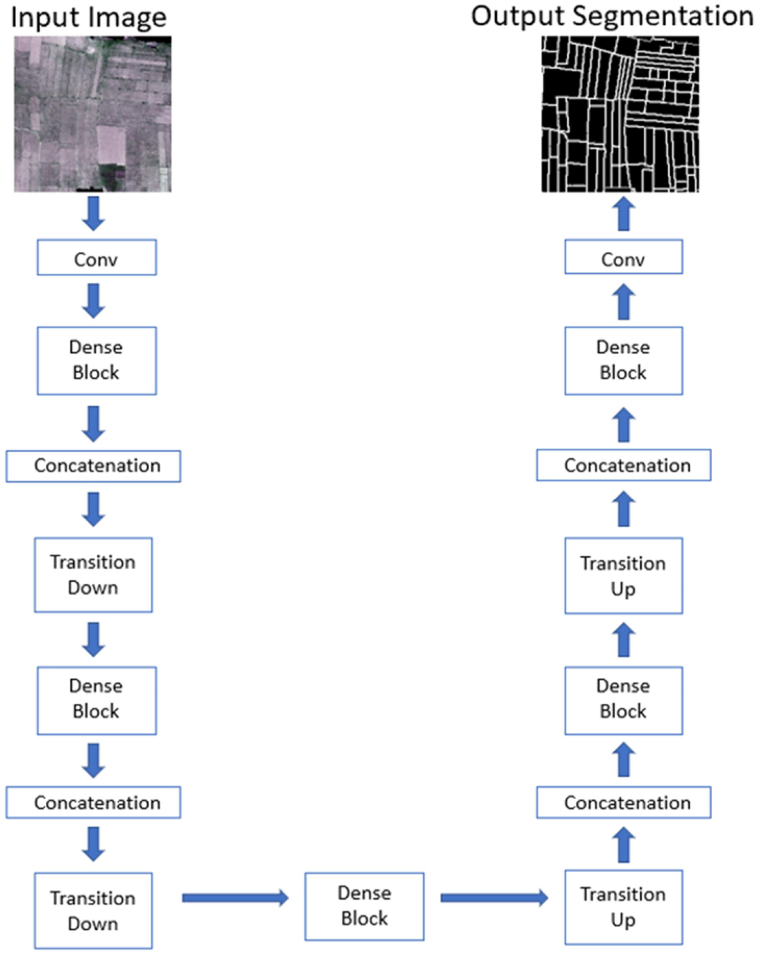
The architecture of the proposed DenseNet model.

**Fig. 7:**
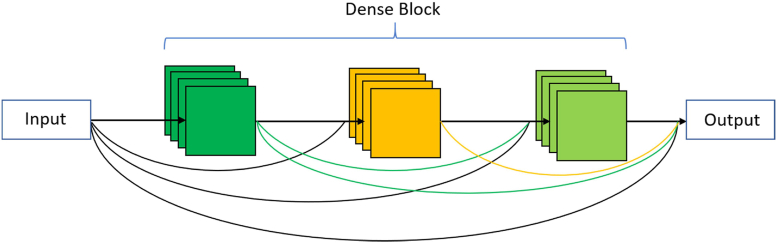
A three-layer dense block. Each layer takes all preceding features as input.

DenseNet (Huang et al., 2017) builds upon ResNet architecture (He et al., 2016) in which each layer connects to every other layer. The DenseNet is built from several dense blocks and other pooling operations like transition down and transition up. In the dense block, each layer concatenates outputs from all preceding layers and passes on its feature-maps to all the subsequent layers. The basic idea of the dense block is to build a dense connection among all previous layers with the later layers. Unlike traditional CNN, the input of each layer is not based on the output of a single layer rather depends on the outputs of all previous layers. The general architecture of DenseNet and dense block are displayed in Fig. 6 and Fig. 7. We used three different depths: 56, 67, and 103 in DenseNet architectures (Table 4) for our field boundary detection problem. The dropout layer is added after the last convolution layer of each dense block to address the overfitting problem. The dropout rate was set to 0.5.

**Table 4:** Parameters of three DenseNet architectures (DB: Dense Block TD: Transition Down TU: Transition Up).

DenseNet56	DenseNet67	DenseNet103
3 × 3 conv layer	3 × 3 conv layer	3 × 3 conv layer
DB (4 layers) +TD	DB (5 layers) +TD	DB (4 layers) + TD
DB (4 layers) +TD	DB (5 layers) +TD	DB (5 layers) +TD
DB (4 layers) +TD	DB (5 layers) +TD	DB (7 layers) +TD
DB (4 layers) +TD	DB (5 layers) +TD	DB (10 layers) +TD
DB (4 layers) +TD	DB (5 layers) +TD	DB (12 layers) +TD
DB (4 layers)	DB (5 layers)	DB (15 layers)
TU + DB (4 layers)	TU + DB (5 layers)	TU + DB (12 layers)
TU + DB (4 layers)	TU + DB (5 layers)	TU + DB (10 layers)
TU + DB (4 layers)	TU + DB (5 layers)	TU + DB (7 layers)
TU + DB (4 layers)	TU + DB (5 layers)	TU + DB (5 layers)
TU + DB (4 layers)	TU + DB (5 layers)	TU + DB (4 layers)
1 × 1 conv (softmax)	1 × 1 conv (softmax)	1 × 1 conv (softmax)

##### 2.4.1.4 FCN-DKConv6

The FCN-DKConv6, with a dilated convolutional layer, which is developed from FCN-DKs (Persello et al., 2017), was used to detect agricultural field boundaries in northern Nigeria ((Musyoka, 2018). The architecture is composed of six convolutional layers followed by batch normalizations and “Leaky Relu” non-linearity. A 1 × 1 convolutional filter is used in the classification layer to predict labels. The details about FCN-DKConv6 are listed in Table 5.

**Table 5:** Final implementation; FCN-DKConv6. BNorm: batch normalization; LRuLu: Leaky ReLu (Musyoka, 2018).

Networks	Layer	weights	Stride	Pad	Dilation
	Conv1	5 × 5 × 8 × 16	1	2	1
FCN-DKConv1	BNorm1	–	1		–
	LReLu1	–	1		–
	Conv2	5 × 5 × 16 × 32	1	4	2
FCN-DKConv2	BNorm2	–	1		–
	LReLu2	–	1		–
	Conv3	5 × 5 × 32 × 32	1	6	3
FCN-DKConv3	BNorm3	–	1		–
	LReLu3	–	1		–
	Conv4	5 × 5 × 32 × 32	1	8	4
FCN-DKConv4	BNorm4	–	1		–
	LReLu4	–	1		–
	Conv5	5 × 5 × 32 × 32	1	10	5
FCN-DKConv5	BNorm5	–	1		–
	LReLu5	–	1		–
	Conv6	5 × 5 × 32 × 32	1	12	6
FCN-DKConv6	BNorm6	–	1		–
	LReLu6 Conv	– 1 × 1 × 32 × 2	1 1		– 1
Classification	Dropout				
	Softmax				

#### 2.4.2 Random forest (RF)

Random forest developed by Breiman (2001), has recently been used for boundary detection in agricultural fields (Debates et al., 2016). The random forest method uses an ensemble of multiple iterations of decision trees where each tree is made by bootstrapping of the original data set. It allows for robust error estimation with the remaining test set, the so-called Out-Of-Bag (OOB) sample. The excluded OOB samples are predicted from the bootstrap samples and by combining the OOB predictions from all trees. We used an RF model with 600 trees and a maximum tree depth of 30 levels of nodes during the training phase. The local binary pattern and co-occurrence matrix features (including contrast, correlation, entropy, and so on) were extracted from the satellite image as the input to train the random forest model.

### 2.5 Training and precision assessment

Before training, a hyper-parameter sensitivity analysis was done for learning rate, weight decay, patch size, sample size, and batch size. The details of the hyper-parameter sensitivity analysis and best parameter selection are listed in Table 6. For each F–CNN model, we selected the hyper-parameter configuration with the highest boundary precision value. We used precision, recall, and F1-score (Dice Similarity Coefficient) (Powers, 2011) to evaluate the model performance. The three metrics can be expressed as follows:

(1) $$\text{Precision}\;=\;\frac{True\;Positive}{True\;Positive+False\;Positive}$$

(2) $$\text{Recall}\;=\;\frac{True\;Positive}{True\;Positive+False\;Negative}$$

(3) $$\text{F}1\;=\;2\times \frac{Precision\times Recall}{Precision+Recall}$$

**Table 6:** Hyper-parameter sensitivity analysis for five F–CNN Models.

Hyper-parameters	Range of Tuning	Best hyper-parameter				
		U-Net	SegNet	Dense56	Dense67	Dense103
Learning Rate	0.01,0.001,0.0001	0.0001	0.0001	0.0001	0.0001	0.0001
Batch Size	1,2,5	1	1	1	1	1
Image Size	100 × 100,192 × 192	192 × 192	192 × 192	192 × 192	192 × 192	192 × 192
Patch Size	3 × 3, 5 × 5, 7 × 7	3 × 3	3 × 3	5 × 5	5 × 5	5 × 5
Dropout Rate	0.2,0.3,0.4,0.5	0.5	0.5	0.5	0.5	0.5

True positive is an outcome where the model correctly predicts the positive class. In our case, this is a boundary pixel that is identified as belonging to the boundary class. False-positive is a non-boundary pixel that is incorrectly identified as a boundary pixel. False-negative is a boundary pixel that is classified as non-boundary. All these parameters can be directly obtained from the confusion matrix in the python “scikit-learn” library (Garreta, 2013).

We applied the dropout, L-2 regularization to address the over-fitting problem. All models were trained with 3-channels (blue, green, and red) and 4-channels (blue, green, red, and NIR2) images. To assess the performance, we used the receiver operating characteristic (ROC) curve and the related area under the ROC curve (AUC).

All networks were trained with the Tensorflow-GPU 1.14.0, CUDA 10.0 toolkit, and cuDNN 7.4 support on a Dell Alienware R8 desktop, which has 16 GB RAM, an 8 GB RTX 2080 super GPU.

## 3 Results

**Fig. 8:**
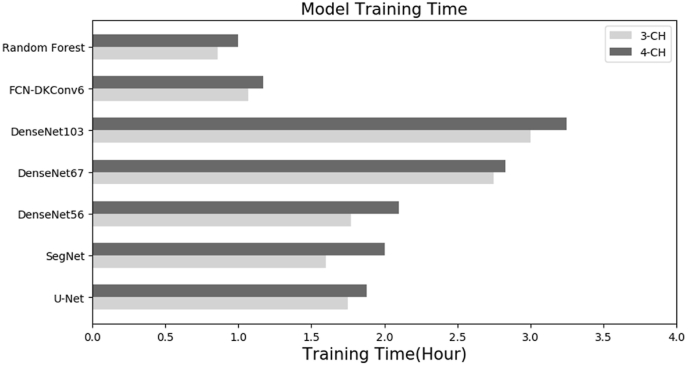
Training times of seven models with 3-channel and 4-channel VHR images.

**Fig. 9:**
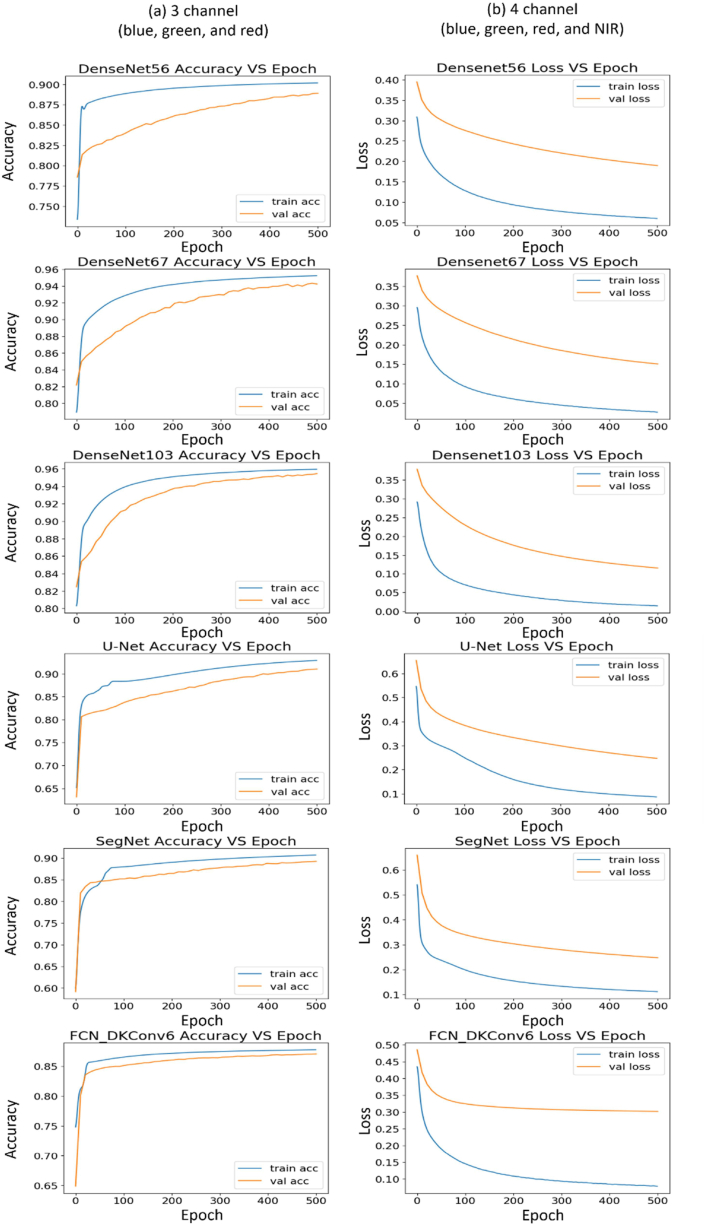
Learning curves of five F–CNN models and FCN-DKConv6 during the training and validation phase.

The training times for the F–CNN architectures and two baseline methods are shown in Fig. 8. For both the 3- and 4-channel images, the random forest model required less training time than all architectures, whereas DenseNet 103 needed the highest training time. On average, the 3- channel images required around 15 min lesser time for training than 4-channel images. Both training and validation log-loss, which is usually related to cross-entropy and measures the performance of a classification model, decreased to the point of stability and showed a small gap between the train and validation loss learning curves (Fig. 9), i.e., low “generalization gap” (Hoffer et al., 2017). The lowest “gaps” were observed for the DenseNet103 architecture indicating the lowest generalization error among all models.

### 3.1 Model performance

During the training stage, all F-CNNs models showed very high recall and F1-scores (>0.80) for both the 3- and 4-channels images (Table 7, Table 8). The highest precision, recall, and F-scores in boundary detection were found with DenseNet103 for both the 3- and 4-channel images (Table 7, Table 8). The lowest values were obtained from the random forest model.

**Table 7:** Precision, recall, F1-scores of five F-CNNs models, and two baseline methods for detecting crop field and boundary from three channels (blue-green-red) high-resolution satellite images.

	Boundary			Cropland		
	Precision	Recall	F1-Score	Precision	Recall	F1-Score
	Training					
Random Forest	0.82	0.63	0.71	0.94	0.98	0.96
FCN-DKConv6	0.85	0.85	0.85	0.96	0.97	0.96
U-Net	0.84	0.85	0.84	0.97	0.97	0.97
SegNet	0.84	0.84	0.84	0.96	0.96	0.96
DenseNet56	0.93	0.93	0.93	0.98	0.98	0.98
DenseNet67	0.93	0.94	0.93	0.99	0.99	0.99
DenseNet103	0.94	0.95	0.94	0.99	0.99	0.99
Test						
Random Forest	0.48	0.21	0.29	0.87	0.96	0.91
FCN-DKConv6	0.56	0.56	0.56	0.92	0.93	0.92
U-Net	0.64	0.65	0.65	0.93	0.93	0.93
SegNet	0.67	0.66	0.66	0.93	0.94	0.94
DenseNet56	0.74	0.74	0.74	0.94	0.95	0.94
DenseNet67	0.77	0.75	0.76	0.95	0.95	0.95
DenseNet103	0.78	0.76	0.77	0.95	0.96	0.96

**Table 8:** Precision, recall, F1-scores of five F–CNN models, and two baseline methods for detecting crop field and boundary from four channels (blue-green-red-NIR2) high-resolution satellite images.

	Boundary			Cropland				
	Precision	Recall	F1-Score	Precision		Recall		F1-Score
	Training							
Random Forest	0.84	0.66	0.74	0.95	0.98		0.96	
FCN-DKConv6	0.88	0.86	0.87	0.95	0.95		0.95	
U-Net	0.83	0.83	0.83	0.97	0.97		0.97	
SegNet	0.84	0.84	0.84	0.97	0.97		0.97	
DenseNet56	0.92	0.91	0.92	0.96	0.96		0.96	
DenseNet67	0.93	0.92	0.92	0.98	0.98		0.98	
DenseNet103	0.94	0.94	0.94	0.99	0.99		0.99	
Test								
Random Forest	0.49	0.22	0.30	0.87	0.96			0.92
FCN-DKConv6	0.61	0.6	0.61	0.91	0.92			0.91
U-Net	0.65	0.65	0.65	0.92	0.92			0.92
SegNet	0.71	0.71	0.71	0.92	0.93			0.92
DenseNet56	0.75	0.74	0.74	0.94	0.94			0.94
DenseNet67	0.77	0.75	0.76	0.94	0.94			0.94
DenseNet103	0.78	0.78	0.78	0.95	0.95			0.95

The algorithms were tested on the hold-out test images to evaluate their performance. The random forest performed very poorly to detected field boundaries among all algorithms, and precisions were only <0.5 for both 3-channel and 4-channel images (Table 7, Table 8). The precision of FCN-DKConv6 dropped from 0.88/0.85 at the training phase to 0.61/0.56 at the testing phase resulted in a very high generalization error (29%) in boundary detection. Wheres, this error was only 15% for DenseNet103, resulted in the highest precision among all tested models in boundary detection from hold-out test images. The precision of two popular F-CNNs models, U-Net and SegNet, was around 0.68. The use of 4-channels images showed a small improvement in precision boundary detection (Table 8).

**Fig. 10:**
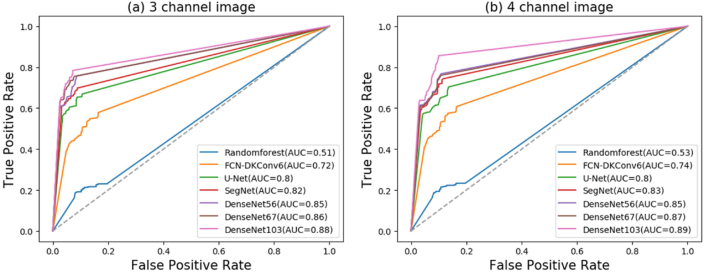
Receiver operating characteristic of five F–CNN models and two baseline methods with (a) 3-channel and (b) 4-channel VHR images.

To further compare the performance of these architectures, we use the area under the ROC curves (AUC) metric. AUC is a standard metric for binary classification tasks that produces a single point in the ROC space. The AUC values < 0.5, 0.7 and >0.9 represent little, moderate and high usefulness of a model for classification tasks, respectively (Swets, 1988). In this study, DenseNet with deeper architecture had the highest AUC values (>0.85) among all models, and the lowest (0.51) was observed in the RF model followed by the FCN-DKConv6 model (Fig. 10). The classification precision and AUC slightly improved with the addition of the NIR2 band for all models.

**Fig. 11:**
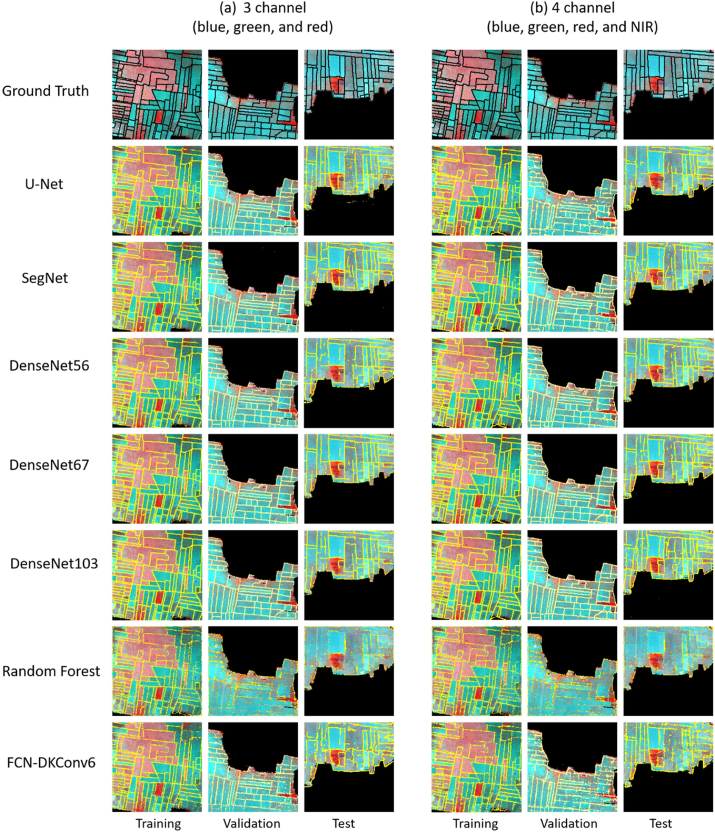
Ground truth and predicted field boundaries from a sample of training, validation, and test images. (a) three channels (blue, green, and red) and (b) four channels (blue, green, red, and NIR) VHR images.

To visualize and compare the predictive performance of all models, predicted field boundaries with corresponding ground truth data of a sample of training, validation, and test images are shown in Fig. 11. The black and yellow lines represent ground truth and predicted field boundaries, respectively. All models except RF were able to detect most of the field boundaries from training and validation images. A significant number of pixels belonging to field boundaries in test images were classified incorrectly by all models. However, compared to five F-CNNs models, a large number of pixels in both 3- and 4-channel images were miss-classified by FCN-DKConv6. The performance of boundary detection by RF was low and unable to detect most of the crop boundary from the test image.

## 4 Discussion

The classification of pixels in an image, also known as semantic classification, is a non-trivial task. Recently, data-driven machine learning methods, especially CNN, have found widespread success in image classification (Kayalibay et al., 2017; Tao et al., 2018; Buslaev et al., 2018). Shallow neural networks (lower number of layers) have a relatively small receptive field that can only learn local characteristics. On the other hand, deep networks have a relatively big receptive field that can learn more abstract features. These learned abstract features are not sensitive to the size, location, and direction of the object and help improve the quality of the recognition performance. Although deep CNN is very successful in object classification, its performance degrades when most of the deep CNN cannot recognize the specific contour of the object, which in our case, is the field boundary.

The F–CNN models used in this study showed very high accuracies for detecting field boundaries. The area under ROC curve values during model evaluation with set-out test images ranged from 0.8 to 0.89. An AUC >0.7 for an algorithm serves as a threshold for usefulness in an application (Swets, 1988). Among all F–CNN models evaluated in this study, the DenseNet 103 showed the highest precision and AUC for detecting boundaries from test images. Since DenseNet includes dense blocks that build dense connections among all previous layers with later layers, it performed better than SegNet and U-Net. Besides, DenseNet is based on implicit deep supervision that can be described as individual layers receiving additional supervision from the loss function through the shorter connections (Lee et al., 2015). It can address the vanishing gradient problem by connecting every layer directly. U-Net and SegNet required less training time for the 3- and 4-channel images than DenseNet, but they were also less precise. As Ronneberger et al. (2015) reported, U-Networks perform reasonably well with limited data sets. SegNet also requires fewer training parameters, which makes it one of the most memory-efficient models (Badrinarayanan et al., 2017). Our tests were performed in a challenging environment, where field boundaries were hard to discern, even by the naked eye. Thus, in less demanding environments, U-Net and SegNet might be good enough to meet the user's requirements.

We compared the performance of all F–CNN models with R-G-B and R-G-B-NIR2 bands to evaluate the NIR band (860–1040 nm) contributions to the classification results. The combination of R-G-B with NIR showed slightly better classification results than the just the R-G-B bands. The result is in agreement with other studies, where the use of multispectral data led to better image classification and segmentation results in computer vision (Gavankar and Ghosh, 2018; Ishii et al., 2016) and remote sensing (Yang et al., 2018a) applications. The encoder-decoder F–CNN architectures performed better than o RF (Debats et al., 2016) and FCN-DKConv6 (Musyoka, 2018) for detecting agricultural field boundary in the smallholder farming system. Different from the proposed encoder-decoder based F–CNN model, the FCN-DKConv6 only consists of a dilated convolutional layer followed by batch normalizations and Leaky Relu non-linearity for the boundary detection. We found inferior predictive performance in RF for detecting the field boundary and acceptable precision (<0.90) of crop field detection. Debats et al. (2016) achieved high performance in detecting crop field by RF across different types of agricultural fields. However, the RF model needs manually extracted features (such as texture) as input for the training. On the contrary, segmentation based CNN models can discover the underlying patterns and automatically works out the most descriptive and salient features related to each image.

The satellite images used in this study were from smallholder agricultural fields in Bangladesh, where the average field size is 0.105 ha with very narrow bunds, typically less than 0.2 m wide, separating the fields. The application of F–CNN based semantic segmentation techniques for detecting crop field boundaries in a complex landscape showed promising results. The precision of all F–CNN methods was around 0.9 during the training and validation stages. When models were evaluated with test images, we got around only 15% lower precision compared to the training stage by DenseNet103, indicating a low generalization error. A FCN-DKConv6 was recently applied by Musyoka (2018) to detect agricultural field boundaries in northern Nigeria, where farmers predominantly grow two or more crops simultaneously in the same field. Moreover, trees are present in nearly all fields. Whereas in Bangladesh, trees are mostly grown on the homesteads of the farmers, rural settlements, and cropland is clustered in separate, yet adjacent regions. Land elevation and drainage largely determine where and when the winter crops can be sown (Krupnik et al., 2017). Therefore, farmers tend to grow their winter crops in clusters, which results in zones with many adjacent fields of the same crop type, which makes the detection of field boundaries even more challenging. Almost similar precision was obtained by different F–CNN techniques to delineate field boundaries in small farms despite a lot of dissimilarities in land use between Nigeria and Bangladesh. The results of our study show the potential of using F–CNN based methods for developing automated field boundary detection algorithms to be used in smallholder farming systems.

## 5 Conclusions

Identification of useful computer vision algorithms is the first step in developing an online application for automatically detecting field boundaries of small-scale farms using imagery from mapping APIs. Results generated by five proposed F–CNN models in this study identified the most effective and accurate algorithms for extracting functional agricultural field boundaries in a complex rice-based cropping system. Even though models were trained with a relatively small size of labeled data, a low generalization error was observed in all F–CNN models. The performance of the outlined F–CNN methods could be further improved with a more extensive set of labeled data. A post-processing technique such as the “Snakes” algorithm that addresses geometric and/or topologic constraints (Kass et al., 1988) needs to be incorporated with a F–CNN model for improving the geometric quality of detected field boundaries.

## CRediT authorship contribution statement

**Ruoyu Yang:** Methodology, Software, Validation, Formal analysis, Data curation, Writing - original draft, Writing - review & editing, Visualization. **Zia U. Ahmed:** Conceptualization, Methodology, Validation, Resources, Writing - original draft, Writing - review & editing, Supervision. **Urs C. Schulthess:** Conceptualization, Writing - review & editing. **Mustafa Kamal:** Investigation, Data curation. **Rahul Rai:** Writing - original draft, Writing - review & editing, Supervision.

## Declaration of competing interest

The authors declare that they have no known competing financial interests or personal relationships that could have appeared to influence the work reported in this paper.
